# *Torulaspora delbrueckii* Phenotypic and Metabolic Profiling towards Its Biotechnological Exploitation

**DOI:** 10.3390/jof8060569

**Published:** 2022-05-26

**Authors:** Flávia Silva-Sousa, Ticiana Fernandes, Fábio Pereira, Diana Rodrigues, Teresa Rito, Carole Camarasa, Ricardo Franco-Duarte, Maria João Sousa

**Affiliations:** 1 CBMA (Centre of Molecular and Environmental Biology), Department of Biology, University of Minho, 4710-057 Braga, Portugal; sousa.s.flavia@gmail.com (F.S.-S.); tixand.tf@gmail.com (T.F.); fabiopereirami148@gmail.com (F.P.); pg37989@alunos.uminho.pt (D.R.); teresarito@bio.uminho.pt (T.R.); 2 Institute of Science and Innovation for Bio-Sustainability (IB-S), University of Minho, 4710-057 Braga, Portugal; 3 SPO, University Montpellier, INRAE, Institut Agro, 34060 Montpellier, France; carole.camarasa@inrae.fr

**Keywords:** non-*Saccharomyces*, wine, bread, biotechnology, phenotypic characterization, metabolic characterization, data mining

## Abstract

Wine is a particularly complex beverage resulting from the combination of several factors, with yeasts being highlighted due to their fundamental role in its development. For many years, non-*Saccharomyces* yeasts were believed to be sources of spoilage and contamination, but this idea was challenged, and many of these yeasts are starting to be explored for their beneficial input to wine character. Among this group, *Torulaspora delbrueckii* is gaining relevance within the wine industry, owing to its low volatile acidity production, increased release of aromatic compounds and enhanced color intensity. In addition, this yeast was also attracting interest in other biotechnological areas, such as bread and beer fermentation. In this work, a set of 40 *T. delbrueckii* strains, of varied geographical and technological origins, was gathered in order to characterize the phenotypic behavior of this species, focusing on different parameters of biotechnological interest. The fermentative performance of the strains was also evaluated through individual fermentations in synthetic grape must with the isolates’ metabolic profile being assessed by HPLC. Data analysis revealed that *T. delbrueckii* growth is significantly affected by high temperature (37 °C) and ethanol concentrations (up to 18%), alongside 1.5 mM SO_2_, showing variable fermentative power and yields. Our computation models suggest that the technological origin of the strains seems to prevail over the geographical origin as regards the influence on yeast properties. The inter-strain variability and profile of the products through the fermentative processes reinforce the potential of *T. delbrueckii* from a biotechnological point of view.

## 1. Introduction

*Torulaspora delbrueckii* was, for a long time, associated with winemaking [1], standing out, today, as one of the most interesting non-*Saccharomyces* yeast species [2,3]. Its use in wine introduces diversity and multiplicity to the standard wine market, which is almost exclusively dominated by *S. cerevisiae*, the yeast par excellence for grape must. In fact, in wine fermentation, either spontaneous or inoculated, the initial steps of the processes are dominated by non-*Saccharomyces* yeasts, leading to the production of higher levels of alcohols derived from the Ehrlich pathway, also to the production of ethyl and acetate esters, in greater yields than those achieved by *Saccharomyces cerevisiae*. Afterwards, these yeasts are gradually replaced by *S. cerevisiae*, which possesses a higher tolerance to ethanol [4,5,6]. Non-*Saccharomyces* yeasts present at the beginning of fermentation were originally considered as the causes of spoilage, due to the association with high levels of unpleasant flavors, but more recent studies revealed that, actually, a great number of those species considerably enhance the sensory profile of wines [7].

At the winemaking industrial level, *T. delbrueckii* attracted attention due to a set of relevant characteristics that brought benefits to the final products, namely, improvements in the aroma profiles correlated with the production of specific fruity esters, terpenes, thiols, higher alcohols and glycerol, together with low acetaldehyde and acetic acid levels [8,9]. In addition, the diversified mouthfeel properties caused by the release of mannoproteins or polysaccharides are an advantage when using this yeast to perform must fermentation, enhancing the sensory perception of the wine [2]. Concerning other industrial uses, *T. delbrueckii* also gathers important biotechnological properties: (a) high production of lactic and succinic acids [10,11], enhancing the applications in the food and beverages’ markets, to produce biodegradable polymers and to potentially replace maleic anhydride [12]; (b) great tolerance to freezing and freeze-thawing, making it suitable for commercial applications in frozen dough formulations [13,14,15]; (c) high osmotic and sulfur dioxide resistance [11,16,17]; (d) in beer fermentation, *T. delbrueckii* influences the aromatic profiles in the brewing process, transforming hip aroma terpenoids and increasing the levels of ethyl hexanoate and ethyl octanoate [18,19]; (e) in mezcal fermenting process, the use of this species showed increased levels of phenyl acetate and of *β*-fructofuranosidase enzymes with fructosyltransferase activity [20,21]; (f) in other fermented products, *T. delbrueckii* allowed the obtaining of a balanced aromatic and fermentative profile, producing a more diversified array of volatile compounds than the ones obtained with *S. cerevisiae* strains, in particular for the production of cocoa [22], tequila [23], cider [24], mead [25] and cheese [26].

In addition, *T. delbrueckii* has genomic features that justifies its advantageous use in industrial applications. Recently, we improved the genome annotation of this species [27], annotating for the first time 32 new proteins in the type strain CBS1146, and revealing particular differences compared to *S. cerevisiae*, connected with metabolism and other functional categories allied with fermentative performance. Furthermore, the recent analysis of the pangenome of this species, achieved using 62 *T. delbrueckii* genomes [28], revealed the existence of five major phylogenetic clades associated with the ecology and geography of the strains. These studies helped to understand the existence of genomic changes associated with the different origins, even though the full phenotypic features associated with them were still lagging behind in terms of characterization and scrutiny.

The phenotypic diversity of large strain collections of *S. cerevisiae* was explored for decades, allowing the choice of promisor strains to enhance the wine’s sensorial attributes [5,29]. The most relevant physiological and phenotypic characteristics of this species, for a commercial use, are high fermentation rates, optimum fermentation temperature, stress resistance (ethanol, osmotic and acidic), killer phenotype, sulfur dioxide (SO_2_) tolerance, hydrogen sulfide (H_2_S) production, glycerol and acetic acid production, synthesis of higher alcohols (e.g., isoamyl alcohol, n-propanol and isobutanol), *β*-galactosidase and proteolytic enzyme activity, copper resistance, foam production and flocculation [30,31]. On the contrary, the phenotypic diversity of *T. delbrueckii* was not yet extensively investigated, despite its increasing interest for industrial applications. Existent studies focused only on a small subset of strains, or on only a few physiological parameters. Ciani and Maccarelli [10] studied the fermentation properties of several non-*Saccharomyces* species, including 90 strains of *T. delbrueckii*, but giving attention to only a small number of the phenotypic traits (fermentation rate, production of ethanol and seven fermentation by-products). In 2009, Renault et al. [32] evaluated the oenological properties of 21 *T. delbrueckii* strains, expanding the current knowledge to the evaluation of major volatile compounds. The authors drew conclusions about the high fermentation capacity associated with this species, combined with the low levels of undesirable volatile compounds produced (in particular, hydrogen sulfide and volatile phenols), low volatile acidity and low glycerol production, in opposition to other non-*Saccharomyces* species. More recently, Escribano et al. [33] evaluated the physico-chemical parameters of the wines fermented with several non-*Saccharomyces* species, and drew conclusions about the abundance of aromatic compounds at the end of the fermentation. Even though this study included the evaluation of a large number of phenotypic traits, only eight strains of *T. delbrueckii* were screened. To the best of our knowledge, no study addressing a large set of *T. delbrueckii* strains and the diversified array of phenotypic and metabolic traits was yet available. This type of screening is mandatory to evaluate the *T. delbrueckii* intra-strain differences and lay the foundations for commercial strain selection programs. The aim of this study was to assess the phenotypic and metabolic technological-relevant features of a large collection of *T. delbrueckii* strains, evaluating their potential, especially for the wine- and bread-making industries.

## 2. Materials and Methods

### 2.1. Strain Collection

A *T. delbrueckii* strain collection was constituted, comprising 40 isolates with diverse geographical origins and belonging to seven technological/ecological groups: wine (11 isolates, 1 of which is a commercial strain); other beverages (2 isolates); bread (4 isolates); food (5 isolates); arboreal/soil (11 isolates); water (3 isolates); clinical (1 isolate) and 3 isolates of an unknown origin (Supplementary Materials, Table S1). All strains were stored at −80 °C in cryotubes containing 1 mL of glycerol (30%, *v*/*v*). Before use, the yeasts were inoculated in YPD plates (yeast extract 1% (*w*/*v*), peptone 2% (*w*/*v*), glucose 2% (*w*/*v*), agar 2% (*w*/*v*)), cultured for 48 h at 30 °C, unless otherwise stated in the text. The genome of all of the strains was already sequenced in our previous work [28], confirming the identification of all of the isolates at the species level.

### 2.2. Phenotypic Characterization

Phenotypic characterization of the *T. delbrueckii* strain collection was performed for a wide range of physiological traits, relevant to the wine and bread-making industries. Two sets of phenotypic tests were performed, the first one based on liquid cultures, and the second one based on solid media. In the first set of phenotypic tests, strains were inoculated into replicate wells of 96-well microplates, following an adaptation of Mendes et al. [30]. Prior to each experiment, the isolates were grown overnight at 30 °C in liquid YPD medium (yeast extract 0.5% (*w*/*v*), peptone 1% (*w*/*v*), glucose 2% (*w*/*v*)). After washing with PBS 1×, the cells were inoculated at a final optical density of 0.1 (OD_640nm_ = 0.1), in quadruplicate, into 96-well microplates containing MS medium [34] supplemented with the appropriate compound requirements to satisfy the different test conditions, as explained further below. The yeast growth was measured considering the values of optical density achieved after 46 h of incubation. This first screening included the following tests: growth at different temperatures (15, 25, 30 and 37 °C) and using different carbon sources (glucose, fructose, maltose, sucrose); ethanol resistance (5, 10, 14 and 18% (*v*/*v*)); tolerance to osmotic, salt and oxidative stresses (1.5 M NaCl, 1 M KCl and 2 mM H_2_O_2_). Growth was also evaluated in the presence of copper sulphate (5 mM CuSO_4_) and the fungicides, fluconazole, myclobutanil, metalaxyl and tebuconazole (0.5 mg/mL).

In a second set of phenotypic tests, *β*-glucosidase activity, hydrogen sulfide (H_2_S) production, ethanol (12% (*v*/*v*)) plus sulfur dioxide (SO_2_) resistance and killer activity were also assessed. All of these tests were performed in solid media, using 5 μL of cell suspensions (OD_640nm_ = 1.0), and subsequently incubating the plates at 30 °C. Although cell suspensions for the *β*-glucosidase activity and killer activity tests were prepared from 48 h-old cultures grown on YEPDA medium, for the other assays they were prepared from an overnight pre-culture in YEPD medium. The *β*-glucosidase activity was tested using two different solid media containing arbutin or esculin as sole carbon sources. The arbutin medium was prepared according to Rosi et al. [35], while the esculin medium was prepared according to the Bile Esculin agar’s manufacturer’s instructions (Merck^®^, Kenilworth, NJ, USA). The cells’ ability to grow and the consequent appearance of a brown color in the colonies, or the appearance of a brown-blackish halo around them, was associated with the production of *β*-glucosidase. The color alteration or the halo formation (halo size) was examined after 2, 4, 6 and 8 days in arbutin plates, or after 2 days in esculin plates. The yeast *S. cerevisiae* (BY4741) and an uninoculated plate were used as negative controls [36].

H_2_S production was evaluated using a bismuth glucose glycine yeast agar medium (BiGGY agar—Fluka^®^, St. Louis, MO, USA; [37]), followed by incubation at 30 °C for 2 days. The color of the colony allowed for the estimation of high (dark brown color), intermediate or low (no color change) producers of H_2_S. Resistance to SO_2_ was evaluated on modified Malt Extract Agar—VegitOne medium (MEA—Millipore^®^, Burlington, MA, USA)—supplemented with 12% (*v*/*v*) of ethanol, and increasing concentrations of SO_2_ (0.25, 0.5, 1.0 and 1.5 mM). Susceptibility phenotypes were registered after incubation at 30 °C for 5 days. Killer activity was measured using the medium described by Buzzini and Martini [38], with some modifications. YEPDA medium containing 0.0015% (*w*/*v*) of methylene blue (MB) and 0.5% (*w*/*v*) of NaCl was adjusted to pH 4.2, with sodium citrate/phosphate buffer. Previously characterized sensitive, killer and neutral *S. cerevisiae* strains were used as the controls. Cell suspension of each strain and the respective controls were plated in two sets of plates (YEPDA-MB), seeded with a sensitive or a killer strain, and the consequent phenotype was registered. The phenotype was considered as sensitive if a blue-colored killing zone appeared in the spot, and it was considered as killer if a blue-colored zone with an inhibition halo appeared around the cells. A negative reaction indicated that the tested cells had a neutral phenotype.

To complete the phenotypic characterization of the *T. delbrueckii* strain collection, the freezing resistance was also assessed, using the protocol described by Alves-Araújo et al. [15]. Briefly, yeast cells were grown on liquid YPS medium (yeast extract 0.2% (*w*/*v*), peptone 2% (*w*/*v*), sucrose, 4% (*w*/*v*), 0.2% (*w*/*v*) KH_2_PO_4_ and 0.1% (*w*/*v*) MgSO_4_.7H_2_O), at 30 °C, until reaching the initial stationary phase (2.4–2.7 × 10^8^ cells/mL). After being harvested, washed and suspended in sterile water to a OD_640nm_ of 12–20, cells were suspended in LF liquid storage medium [39], formulated to simulate bread dough. Samples were frozen at −20 °C for different time periods (15, 30, 80 and 120 days). Afterwards, at each time point a sample was thawed, and serial diluted, spotted onto the YEPDA plates, and the colony-forming units (CFU) were counted after 48 h of growth at 30 °C.

All phenotypic results were assigned to a class between 0 and 3, to facilitate the mathematical data comparison between different experimental datasets, as previously described [30]. In detail, the growth was scored in the following way, both in liquid and solid media: score 0 represents no growth (DO_640_ = 0.1) or no visible growth on solid media; score 3 represents at least 1.5-fold increase of DO_640_, and extensive growth on solid media; scores 1 and 2 indicate intermediate values. For the change of color in arbutin and BiGGY media: score 0 corresponds to no visual growth after 2 or 8 days; score 3 corresponds to dark brown or brown color colonies, and scores 1 and 2 to color colonies ranging from white to brown. The halo size obtained in the esculin medium was categorized in the following way: score 0 was attributed to colonies that did not show a halo; score 3 to colonies with a halo size higher than 0.6 cm, and scores 1 and 2 to halo sizes below or equal to 0.4 cm (*S. cerevisiae* control) or to a halo size between 0.4 and 0.6 cm, respectively. Cell viability after freezing, in a similar way, was categorized using the following scores: score 0 stands for cell viability < 50% after 15 days; score 3: cell viability > 50% after 120 days; scores 1 and 2: cell viability < 50% after 30 days, or <50% after 80 and 120 days. Finally, for the killer activity assessment, binary attribution was used, scoring 1 in the case of the existence of a killer or sensitive phenotype, and 0 when its absence is noticed (neutral strains).

### 2.3. Individual Fermentations and Metabolites Quantification

Each *T. delbrueckii* strain was subjected to individual fermentations, in triplicate, using MS medium (ratio of 1:2 of medium to void volume), at 18 °C, according to Franco-Duarte et al. [5]. Fermentation samples were collected after 196 h, and treated with perchloric acid (2% (*v*/*v*)) on ice for 30 min, for deproteinization. After centrifugation at 12,000× *g* for 10 min, supernatant samples were filtered through a 0.22 μm pore filter, and used for further analysis.

The fermentative profile of *T. delbrueckii* strains was evaluated using a high-performance liquid chromatography (HPLC) apparatus, equipped with carbohydrate H^+^ 9 μm HyperRez XP column (Thermo Scientific™, Waltham, MA, USA), in order to quantify the ethanol, sugar (glucose), glycerol and organic acids (acetic, citric, formic, succinic acids). The column’s temperature was 40 °C and the flow rate was 0.5 mL/min, constant during the race time of 30 min. The metabolites were identified through their relative retention times, compared with the respective standards. To determine the concentration of the detected compounds, arabinose (20 g/L) was used as the internal standard. Chromeleon 7.2.9 software was used for data collection.

### 2.4. Data Analysis Using Statistical and Data Mining Methods

The phenotypic and metabolic inter-strain variability of *T. delbrueckii* was evaluated by principal component analysis (PCA), available in the Orange data mining suite software v. 3.25.0 [40]. A set of standard predictive data-mining methods were used for the inference of prediction models. Data analysis was performed in the Orange software, and validated in python, using the scikit learn package. In particular, naïve Bayes, decision tress, support vector machines (SVM), k-nearest-neighbor (kNN) classifier, random forest, stochastic gradient descent (SGD), CN2 rule inducer and neural networks were used to predict the technological groups of the 40 *T. delbrueckii* strains, using both phenotypic and metabolic datasets. After 10-fold cross validation of the training data, performance scores were assessed, in particular the area under the receiver operating characteristics (ROC) curve (AUC) [41], that estimates the probability that the predictive model would correctly differentiate between distinct technological applications, given the associated pairs of strains. Based on the performance scores, neural networks were selected as the best classifiers, and used to build a confusion matrix comparing the predicted groups with the real ones.

## 3. Results and Discussion

### 3.1. T. delbrueckii Intra-Strain Phenotypic Heterogeneity

Our set of *T. delbrueckii* strains included 40 isolates from different geographical and technological applications (Supplementary Materials, Table S1). Strains were categorized into seven different technological/ecological groups, according to their origins: wine (1 commercial wine strain and 10 isolates obtained from winemaking environments); other beverages (2 isolates); bread (4 isolates); food (5 isolates from vegetables, fruit and dairy products); arboreal/soil (11 isolates from vascular plants, leaves and soil); water (3 isolates from river and sea waters); clinical (1 isolate) and 3 isolates with unknown origin. Phenotypic screening of this entire collection was performed to evaluate the strain-specific patterns, using a battery of phenotypic tests with potential biotechnological interest, especially for the wine and bread-making industries. As presented in Table 1, the phenotypic results revealed a substantial phenotypic variation among the entire *T. delbrueckii* strain collection. This is, to the best of our knowledge, the largest phenotypic characterization of a *T. delbrueckii* strain subset.

The response of the *T. delbrueckii* strains to different conditions was first assessed by monitoring their growth in 96-well microplates using liquid MS medium, simulating grape must, as previously described by Mendes et al. [30]. The phenotypic tests included growth at a vast range of temperatures and in different carbon sources, as well as in the presence of fungicides, ethanol, and under osmotic, salt and oxidative stresses. According to the results (expressed by growth performance, graded from 0 to 3), a large number of the yeasts displayed an absence of growth at 37 °C, (class 0), showing that this temperature is already extreme for *T. delbrueckii*. On the other hand, the temperature range between 25–30 °C appears to be the optimum range for the growth of *T. delbrueckii*, since a large number of strains were labelled in class 3 under these conditions. According to the literature, the optimum growth temperature for *S. cerevisiae* is within the range of 30–35 °C [42]. The ideal temperature for *T. delbrueckii*, according to the results obtained in this work, and in agreement with what was found for one *T. delbrueckii* strain in another study, appeared to be between 25 and 30 °C. In this way, the optimum temperature for *T. delbrueckii* seems to be slightly lower than the one reported for *S. cerevisiae* [43]. Nonetheless, the ability to grow under lower temperatures can be an advantageous feature for the manufacture of specific wines, such as rosé and white wines, whose conditions contribute to an increase in the production of volatile compounds, alongside a fresh character during yeast fermentation [44]. As shown in Table 1, some strains exhibited good growth capacity at 15 °C (eight strains achieved class 3—DO_640nm_ > 1.0, and 65% were categorized in class 2), which could be applied in the aforementioned wine styles. This finding suggests a phenotypic adaptation, similar to that reported for *S. cerevisiae*, which synthesizes the proteins involved in the response to low temperatures [45].

As expected, cell growth decreased drastically with increasing concentrations of ethanol, except for the concentration of 5% (*v*/*v*), in which 36 strains (90% of the screened collection) were attributed to class 3 (Table 1), which indicate that above this concentration cell growth was severely affected. *T. delbrueckii* strains evidenced metabolic flexibility, being able to process all of the tested carbon sources, a relevant feature both in the wine- and bread-making industries. However, while all of the strains were able to metabolize glucose, fructose and sucrose (100% of the strains reached at least class 2, >95% of the strains in class 3), the results regarding the cells’ ability to process maltose demonstrated greater heterogeneity, as only 80% of the strains were categorized in classes 2 or 3. The *T. delbrueckii* strains appeared to have less preference for maltose, in comparison with the other tested carbon sources. This differential preference was revealed to be statistically significant (Supplementary Materials, Figure S1), leading to the conclusion of a similar phenotypic behavior between *T. delbrueckii* and reports on *S. cerevisiae* [46], as both grew in the presence of glucose, fructose, sucrose and maltose, but revealed less preference for maltose degradation. However, all of the *T. delbrueckii* strains showed at least residual growth (duplication of the initial optical density; class 1) in maltose, which indicates that this carbon source could be metabolized by all of the *T. delbrueckii* strains tested. This is of particular importance since maltose is a compound found in some wines [47] and is a major substrate in bread dough fermentation [48]. When comparing our results with the assimilation tests described by Kurtzman et al. [49] for the physiological description of the species *T. delbrueckii*, we confirmed slow/weak growth at 37 °C as a particular feature of this species, and also a strong capacity to assimilate glucose.

Particular differences were, however, observed, regarding some of the tests. First, the assimilation of sucrose revealed that 100% of the strains were able to assimilate this carbon source, even though Kurtzman et al. reported a variable pattern. Capacity to grow in the presence of NaCl was reported as positive by Kurtzman et al., but our strains showed a variable pattern, which could be justified by a difference in the concentrations tested in both works. The ability to assimilate maltose as a carbon source was classified as variable by Kurtzman et al., even though in our work all of the strains were able to use it for growth, however, with different rates.

Despite presenting different structures, glucose and fructose share the same empirical formula (C_6_H_12_O_6_), and were previously shown to be transported by *T. delbrueckii*, across the cell membrane through common carriers, enabling the yeast to use both fermentable sugars in the wine must [50,51]. When available simultaneously in the media, glucose is usually used at a faster rate than fructose, due to the higher affinity of the transporters for glucose [52]. When used as sole carbon sources, however, comparison of growth was not significantly different with fructose or glucose (Supplementary Materials, Figure S1 and Table 1). Sucrose, a disaccharide, can be hydrolyzed by *S. cerevisiae* into one molecule of glucose and another of fructose, through an extracellular invertase present in this yeast, which enables the separation into these monosaccharides [53]. *T. delbrueckii* invertase activity was also described in a baker’s yeast strain [54], and seems to be generalized to this species, enabling growth in MS medium supplemented with sucrose.

Strains’ growth when exposed to stressful conditions revealed a wide heterogeneity associated with this yeast species. In the presence of NaCl (1.5 M), an osmotic stress inducer, strains were distributed throughout all of the classes, with the majority centered at class 2 (and only 5% of the strains unable to grow), while under saline stress mimicked by the presence of KCl (1 M), almost all of the cells reached class 3 of growth. Overall, our results on *T. delbrueckii* revealed a good ability to grow in the presence of NaCl and KCl, which is in agreement with Hernandez, Prieto & Randez-Gil [14], hypothesizing this capacity as a strain-dependent feature. It was noteworthy that the NaCl test contributed greater strain heterogeneity than KCl, which was corroborated by the same authors, who highlighted a more toxic effect of NaCl than KCl, in equivalent concentrations. In the presence of other stress-inducing compounds, such as hydrogen peroxide (2 mM) and copper (5 mM), the yeasts revealed a high variability, with some being completely inhibited by these compounds (32.5% and 47.5% of strains, respectively, categorized in class 0). Yeasts exposed to stressful factors undergo modifications at distinct levels, from metabolic and gene expression alterations, to morphological and behavioral changes, which can interfere with the fermentation process [55,56]. Nonetheless, these adaptations can lead to strain diversity, as some species, namely *S. cerevisiae*, develop specific control mechanisms that trigger responses, as, for example, the activation of the HOG pathway, in order to survive. In this context, a balance is required by the cell once the overactivation of the aforesaid pathway could be lethal, while the lack of its activation turns the cells osmo-sensitive [57]. In this context, the adaptation process may be associated to common defense mechanisms in these strains, in contrast to those with growth difficulties. Regarding other stress-inducing compounds such as copper (CuSO_4_) and H_2_O_2_ (oxidative stress), a large behavioral diversity of the *T. delbrueckii* strains was observed. Scarce information is available regarding the adaptation of *T. delbrueckii* to these chemicals. However, the works of Budroni et al. [58] and Capece et al. [59] on *S. cerevisiae* flor yeasts, revealed that their ability to create films on the surface of wine is related to their resistance to copper, to oxidative stress and even to ethanol resistance. Thus, the observed *T. delbrueckii* specific adaptation, when subjected to these factors, deserves additional work.

Additionally, most strains revealed the ability to grow in the presence of the fungicides myclobutanil, metalaxyl, tebuconazole and fluconazole; all were tested at a concentration of 0.5 mg/mL, although with differentiated growth performance. In detail, metalaxyl revealed to be completely innocuous to *T. delbrueckii* at the concentration tested (100% of strains categorized in class 3), while myclobutanil was the one with less strains achieving higher growth categories. These results about fungicides’ resistance are of great importance since the control of undesirable microorganisms and diseases, through the application of fungicides and pesticides, is one of the major goals to protect wine production nowadays [60]. In addition, chemical fungicides which are usually not specific for pests can lead to the presence of organic residues in the vineyards, which remain in the must, affecting the biological function of other microorganisms, including those used in the fermentation of food and beverages [61]. However, their effect on the growth of *T. delbrueckii* was until now unknown, at least in great detail. As reported in the literature, metalaxyl is one of the fungicides that display an inhibitory effect on the growth of fungi [62]. However, our results showed no effect of this fungicide on the growth or viability of *T. delbrueckii* at the concentration of 0.5 mg/L, a value, in fact, higher than the one tested by Wang et al. [62], which could represent a low sensitivity of this species to this particular fungicide.

The maximum residue level limit for metalaxyl present in wine grapes is currently set by the European Union at 1 mg/L, and Russo et al. [61] report *T. delbrueckii* as having a higher resistance than strains of *S. cerevisiae*, when inoculated in medium supplemented with a mixture of metalaxyl and folpet. Fluconazole, myclobutanil and tebuconazole induced inhibition of *T. delbrueckii* cell growth (Table 1), which is in accordance with the reports by François et al. [63]. However, since only one concentration (0.5 mg/L) was tested for all four of the fungicides, we cannot exclude that metalaxyl could have an effect for higher concentrations, at least for some strains.

The second set of tests included in the phenotypic screening was performed in solid culture media and consisted of the evaluation of *β*-glucosidase activity, hydrogen sulfide (H_2_S) production, sulfur dioxide (SO_2_) resistance and killer activity (Table 1). The *β*-glucosidase production, translated into the potential ability to improve wine aroma profiles [64], was assessed by growing the cells in two different solid media, containing arbutin and esculin as sole carbon sources. Results showed high heterogeneity in both media, since the strains were distributed across all of the classes, even though most strains were assigned to classes 1 (48% for arbutin and 30% for esculin media) and 2 (45% for arbutin and 60% for esculin media). *T. delbrueckii* is not commonly known as a *β*-galactosidase producer, even though some reports have already described that some strains demonstrate efficiency in fermenting lactose, concluding about the presence of *β*-galactosidase [65,66].

Evaluation of hydrogen sulfide (H_2_S) production, an undesirable metabolite associated with off-flavors [67], demonstrated that most *T. delbrueckii* isolates appeared to produce intermediate (class 2–25% of the strains), to high amounts (class 3–60% of the strains) of H_2_S. This production was evaluated using BiGGY agar medium, containing bismuth sulfite, which reacts with H_2_S to form a brown precipitate, which allows a distinction to be made between colonies using their color: the higher the H_2_S production, the darker their colonies appear. This variation in color also reflects differences in sulfite reductase activity, as shown by Jiranek et al. [37]. The *T. delbrueckii* strains tested in the present work showed a high capacity to produce H_2_S, with 60% of the strain collection achieving the higher phenotypic category. These results are opposed to what was known so far regarding this species, since in the work of Renault et al. [32], no strain achieved the higher level of production, with almost all of the strains categorized as average producers. However, since the strains used as controls were not the same, the results could not be properly compared.

Additionally, the phenotypic results regarding the resistance to sulfur dioxide (SO_2_), an agent widely applied in the wine industry [68], evidenced a marked distribution between all of the phenotypic classes. This compound is used as a preservative in oenology, serving as an antioxidant, antimicrobial and anti-oxidasic [69]. As expected, there was a direct correlation between the increasing SO_2_ concentrations and the growth inhibition of the yeast, but even despite the observed susceptibility, some strains were able to grow at the higher concentration of SO_2_ (1.5 mM). Since this test combines double resistance to SO_2_ and ethanol at 12% (*v*/*v*), we should analyze that some differences were observed regarding the strains’ resistance to ethanol, when comparing with results obtained using a liquid synthetic medium. We hypothesize that some ethanol evaporation could help to justify the differences observed in the tests, even though we believe the influence should be the same for all of the strains when comparing the same batch of tests. The high resistance of *T. delbrueckii* strains to sulfur dioxide, detected in the present study, is shared with other species (*S. cerevisiae*, *Saccharomycodes ludwigii*, *Hanseniaspora* sp., *Zygosaccharomyces bailii*), even though others are more sensitive (*Kloeckera apiculate* and *Hansenula anomala*) [68]. These results highlight the importance of using *T. delbrueckii* to ferment musts with higher presence of SO_2_, thus preventing the growth of spoiler species such as *Brettanomyces* spp.

The results presented in Table 1, indicate that only one strain in the *T. delbrueckii* entire collection had a killer activity against previously characterized *S. cerevisiae* strains, used as controls. In contrast, four strains presented a sensitive phenotype against the killer *S. cerevisiae* strain. The finding of one *T. delbrueckii* killer yeast could represent an important result for the winemaking industries, since this isolate could kill the omnipresent *Saccharomyces* yeasts, during fermentation, improving the quality of wine by bringing the advantages associated with *T. delbrueckii* fermentations. Isolation of the *T. delbrueckii* killer strains was already reported before [70], although not in the depth usually found for *S. cerevisiae*. In addition, the results of Velásquez et al. [71] and Ramírez et al. [72] show the isolation of new *T. delbrueckii* killer strains, that dominated and completed the must fermentation, obtaining values above 11% of ethanol, in opposition to the non-killer strains.

Tolerance to freezing stress conditions, perceived by the preservation of cell viability over 120 days at −20 °C, also revealed great phenotypic variability. Nevertheless, only seven strains did not demonstrate any resistance to freezing. Our results are in accordance with the ones obtained by Alves-Araújo [15] showing that the *T. delbrueckii* strain PYCC5323 did not lose cell viability for at least 120 days at −20 °C. However, in the mentioned work, *S. cerevisiae* baker’s yeast lost 80% of its viability in these conditions, after 15 days. Our baker’s strains, on the contrary, showed no loss of viability in the assay, with results showing more than 50% of cell viability after the 120 days of freezing (class 3 of growth) for these strains. Furthermore, the resistance to freezing seems to be a more generalized phenotype in this species, as most stains (67.5%) were graded in class 2 or 3 for this feature.

### 3.2. Principal Component Analysis Evaluation of Phenotypic Variation

The global profile of intra-strain phenotypic variation was evaluated using principal component analysis (PCA) of the data generated in the screening approach, carried out considering 31 phenotypic tests from a set of 40 strains. From the totality of the 31 phenotypic tests performed, in order to avoid overfeeding of the statistical models, only a single concentration of ethanol and of SO_2_ were considered in the PCA. In particular, a preliminary test was performed to evaluate the contribution of each test for the global PCA heterogeneity, and only the ones with higher relevance were considered. However, identical results would be obtained with the remaining ethanol and SO_2_ concentrations. In this way, a total of 24 of the most relevant tests were considered in the PCA analysis.

**Figure 1:**
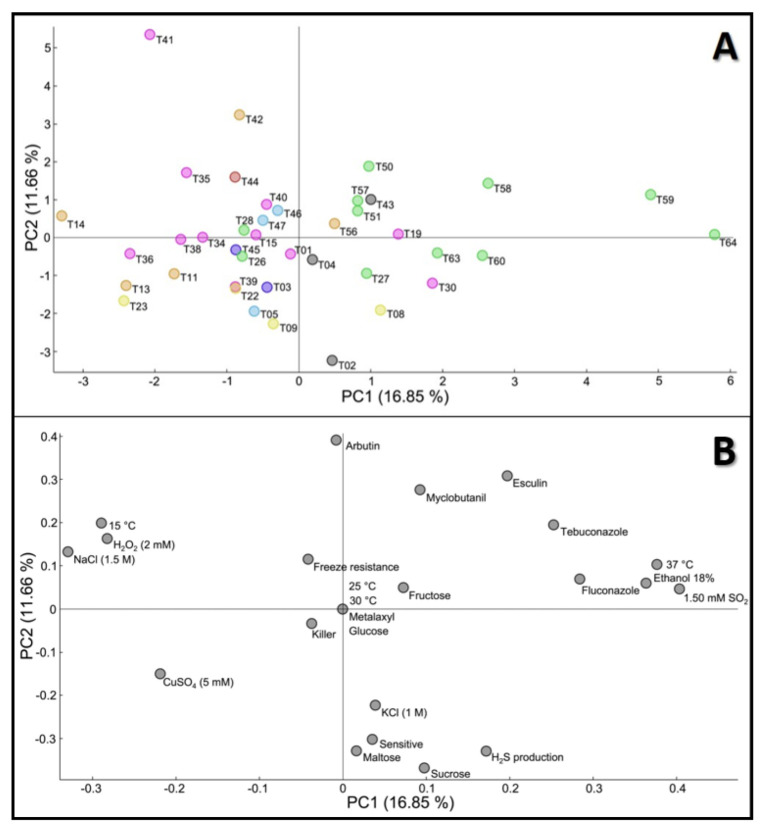
Principal component analysis of phenotypic data of 40 *T. delbrueckii* strains. (**A**) Scores—40 strains distribution. Colors represent the technological application or origin of the strains: ●—winemaking; ●—arboreal/soil; ●—food; ●—bread; ●—water; ●—clinical; ●—other beverages; ●—unknown origin; (**B**) Loadings—24 phenotypic tests.

PCA results showed the segregation of all of the isolates (Figure 1A—scores) and the phenotypic variables (Figure 1B—loadings), in the first two PCA components. Strain variability is explained by the first two principal components, up to a total of 28.6% (PC1—16.9% and PC2—11.7%). The visualization using PC1 and PC3 is presented in the Supplementary Materials, Figure S2, and explains a total of 27.5% of strain variability (PC1—16.9% and PC3—10.6%). Each strain was categorized according to their technologic group, in order to relate them to their phenotypic behavior. Although all of the variables contributed to the diversity of the species, the phenotypes responsible for the highest diversity observed between the strains appear to be related to the presence of SO_2_, in the presence of high ethanol concentration (18% (*v*/*v*)), and the use of high temperatures (37 °C), all discriminated by the first component (the one explaining the higher percentage of variability). The second component (PC2, highlighting 11.7% of variability), showed a marked influence mainly by the *β*-galactosidase activity evaluated on the arbutin medium, the capacity to assimilate sucrose and maltose, and also by the capacity to produce H_2_S and the sensitive phenotype in the killer test (Figure 1B).

As displayed in Figure 1A, PCA allowed the visualization of some clear patterns between strains sharing the same origin. A separation, not total but representative, of arboreal/soil isolates (●) was obtained within the first component (PC1), with these strains accumulating mainly in the right part of the PCA. These results indicate a marked influence of these strains by the presence of high ethanol concentrations and SO_2_ in the media (higher resistance), by the growth at 37 °C (higher resistance) and by the effect, although to a lesser extent, of NaCl, H_2_O_2_ and 15 °C (lower resistance). The opposite was observed for the yeasts from the other groups, in particular for the strains from food (●); bread (●); water (●); clinical (●); other beverages (●) and unknown origins (●), that were mostly concentrated in the left quadrants of the PCA.

The same type of separation, mainly between arboreal/soil and winemaking strains, was also validated by the heatmap clustering, as shown in the Supplementary Materials, Figure S3. The group of wine strains (●) showed a slight dispersion throughout all of the quadrants of the PCA, even though they accumulated mainly in the left part of the PCA. The largest exception was strain T30, the only commercial strain in the collection, which stood out from almost all of the remaining ones, locating near the arboreal/soil isolates, under the influence of PC1 (Figure 1A). The positioning of this commercial strain, apart from the remaining natural wine strains, points to particular phenotypic features of this isolate, highlighting their ethanol and SO_2_ resistance, together with a higher capacity to grow at 37 °C, characteristics that are important for its commercial use. Strains isolated from water (●), other beverages (●) and unknown (●) groups, also show particular clusters in the left part of the PCA. However, a higher number of the strains from these groups should be studied, to establish more robust correlations. In a similar way, the only clinical isolate considered in this study, (●), was located in the upper central part of the PCA, but due to the unrepresentative number of strains from this group, further analysis will be needed before drawing any conclusions.

### 3.3. Metabolic Characterization

**Figure 2:**
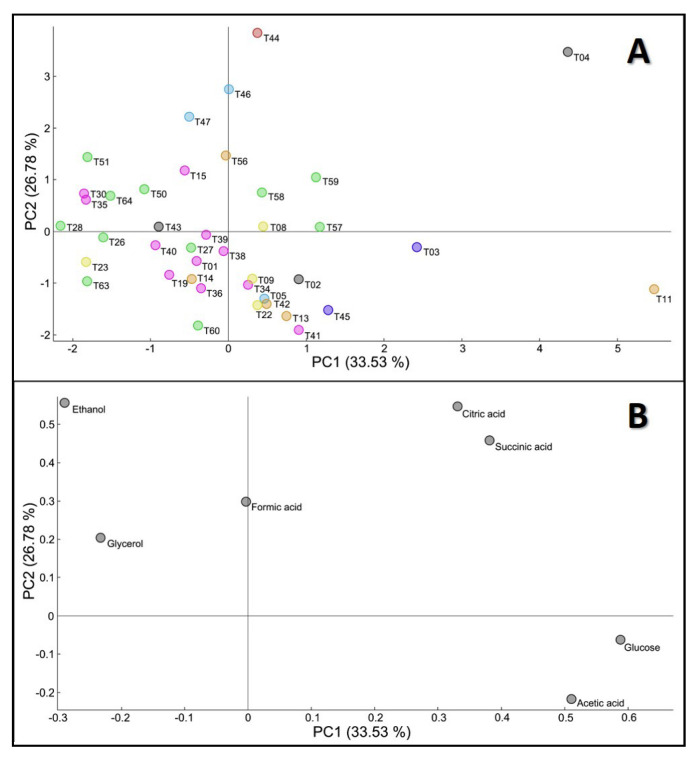
Principal component analysis of metabolic data of 40 *T. delbrueckii* strains. (**A**) Scores—40 strains distribution. Colors represent the technological application or origin of the strains: ●—winemaking; ●—arboreal/soil; ●—food; ●—bread; ●—water; ●—clinical; ●—other beverages; ●—unknown origin; (**B**) Loadings—concentration of seven metabolites obtained by HPLC analysis, after 192 h of fermentation.

The metabolic profile of *T. delbrueckii* was evaluated by HPLC quantification of supernatants collected after 192 h of individual fermentations, performed in MS medium at 18 °C. Inter-strain differences were observed regarding the production and consumption of different compounds, such as glucose, ethanol, organic acids (citric, succinic, acetic and formic) and glycerol (Supplementary Materials, Figure S4). PCA visualization of metabolic data (Figure 2), obtained after HPLC analysis of samples from the end of fermentation, explained approximately 62% of strain variability in the first two components (PC1—33.53% and PC2—26.78%; PC1 versus PC3 represented in the Supplementary Materials, Figure S5). This analysis showed that strain variance was mainly influenced by glucose and acetic acid concentrations at the end of the fermentative process, but also by the quantification of ethanol and citric and succinic acids (Figure 2B). Formic acid, due to its almost residual production by *T. delbrueckii* strains, was placed in a neutral position in the center of the PCA, without any contribution to explain the strain variability.

Overall, even though HPLC analysis revealed that the inter-strain variability at 192 h of fermentation was higher than the inter-group differences, partial strain stratification was observed in the PCA, in accordance with their technological group (Figure 2A); the majority of wine (●) and arboreal/soil isolates (●) showed a tendency to be located on the left part of the figure, under the influence of the first component, explained by low levels of glucose and high production of ethanol, at the end of the fermentation. This segregation highlights a greater fermentative capacity of these isolates, in particular for strains T28, T63, T26, T35, T51 and T30, the latter being the commercial wine strain. From these sub-group of strains, only strains T30 and T35 belong to the wine category. Our hypothesis is that the remaining strains from this sub-group could have gained adaptation mechanisms, under the influence of particular environmental constraints, that increased their potential to be used in winemaking. Similar scenarios were already observed in other yeast species [73,74,75].

On the contrary, strains originating from food (●), bread (●) and other beverages (●) are mostly spread in the lower part of the PCA, under the influence of PC2, this being justified by a possible increased production of acetic acid and lower fermentative power (less ethanol produced and high levels of residual glucose at the end of the fermentation), together also with a low production of citric acid. Strains from unknown origin (●) did not group in a well-defined cluster, being dispersed throughout the PCA, as expected, since they could have multiple origins. The *T. delbrueckii* type strain (T04) appeared detached from the other strains mainly due to low glucose consumption and ethanol production, which translated into poor fermentative power. A similar scenario was obtained before with *S. cerevisiae*, in which the laboratorial type strain showed reduced fermentative potential and lower production of aromatic and volatile compounds [31].

In fact, for almost all of the *T. delbrueckii* strains tested, a correlation between glucose consumption and ethanol production was observed at 192 h of fermentation, as strains with the greatest residual glucose contents (designated as reduced fermentative capacity) translated in lower levels of ethanol production, while those with higher fermentative power released superior concentrations of ethanol, as validated by the extreme positioning of these two variables in the PCA of Figure 2. The strain T11 (located at the far right of Figure 2A) showed the lowest fermentation yield, which could be correlated with its origin from food.

Our results are corroborated by the ones obtained by Alves-Araújo et al. [54], that showed a similar fermentative behavior between *T. delbrueckii* and *S. cerevisiae*, with regard to sugar utilization and regulatory patterns, and slightly lower ethanol yields. The *T. delbrueckii* species is usually defined as a low ethanol producer [2,25,76], however in the work of Escribano et al. [33], *T. delbrueckii* was also one of the highest ethanol producers, among the non-*Saccharomyces* species tested. Additionally, Canonico et al. [77] reported ethanol production in values higher than 12% (*v*/*v*) by *T. delbrueckii* strains, and Catrileo et al. [78], using adaptive laboratory evolution, were able to obtain a strain capable of producing ethanol levels of 11.5% (*v*/*v*). In this way, we concluded that this characteristic is strain-dependent, and should not be attributed as a feature associated to the species.

Concentration of organic acids (acetic, succinic and citric) explained a considerable part of the strain variability observed in this study at 192 h of fermentation (Figure 2 and Supplementary Materials, Figure S4). Strains T11 (obtained from food), T04 (the type strain), strains T57, T58 and T59 (the arboreal strains) revealed variable patterns in terms of the final concentration of these acids at the end of fermentation, not related to the average behavior within their technological group. The importance of these results is justified by the fact that the complexity of wines frequently arises due to the presence of these acids, as they have a direct influence not only on the color, taste and balance of the final product but also on the protection from bacteria that may exist in the environment.

Citric acid contributes to the wine aroma profile with a pleasant citrus-like taste [79]. Liu et al. [80] carried out individual fermentations for *S. cerevisiae* and *T. delbrueckii* and found similarities between both species regarding citric acid yields, as *S. cerevisiae* reached concentrations of 2.23 g/L and the two *T. delbrueckii* strains ranged from 2.18 to 2.36 g/L of citric acid produced. In the present work, no association between the strains’ origin and their citric acid production could be drawn.

Regarding succinic acid, our results evidenced a great heterogeneity of production at 192 h of fermentation, with concentrations ranging from 0.05 to 1.12 g/L, with the highest value being achieved, surprisingly, by the type strain. Our results are in agreement with the literature and with the ones obtained for *S. cerevisiae*. In fact, Franco-Duarte et al. [81], using natural isolates of *S. cerevisiae*, attained a maximum concentration of 1.13 g/L of succinic acid, a value equivalent to the one obtained in this study with *T. delbrueckii*. Puertas et al. [82] also reported *T. delbrueckii* to be a higher succinic acid producer, with measured concentrations ranging between 0.84 and 1.11 g/L, in comparison with *S. cerevisiae* that only reached maximum values of 0.65 g/L in the same study. Findings by Contreras et al. [83] and Liu et al. [80] reinforced this statement, by describing values greater than 1 g/L of succinic for *T. delbrueckii,* depending on the availability of oxygen during the fermentation process.

HPLC analysis emphasized a reverse contribution of succinic and acetic acid, which is in agreement with the reported effects on the fermentation yields for *S. cerevisiae* [81,84,85]. Acetic acid, a relevant end-product of fermentation, represents a key signature in the volatile acidity of wines and, therefore, in its aroma profile. However, excessive concentrations of this chemical are highly detrimental to the quality of the wine. *S. cerevisiae’s* ability to produce acetic acid is described as strain-dependent [86], with values ranging between 0.25–0.50 g/L, under specific fermentative conditions, but it may be greater in response to high-sugar levels, reaching up to 1.8 g/L or even higher [87]. On the other hand, *T. delbrueckii* is reported in the literature as a lower acetic acid producer [3,33,88], in comparison with *S. cerevisiae.* Our results show a great variability in what regards acetic acid production, as the lowest value of acetic acid detected at the end of *T. delbrueckii* fermentations was 0.01 g/L, associated with a strain from arboreal/soil origin, but the highest concentration achieved was 4.64 g/L, obtained by a strain from food ecology, which is far from the values reported for this species.

One final assessment regarding *T. delbrueckii’s* ability to produce acids concerns formic acid. Only three strains were able to produce this compound at the final stage of fermentation, in contents ranging from 0.13 to 0.22 g/L, revealing a poor or almost non-existent capacity of this species to release formic acid. Due to the limited information on the production *by T. delbrueckii* of this acid, to the best of our knowledge, no studies were found for this non-*Saccharomyces* species, and further evaluations should be used in the future to clarify it.

### 3.4. Bioinformatic Prediction of T. delbrueckii Biotechnological Potential

**Figure 3:**
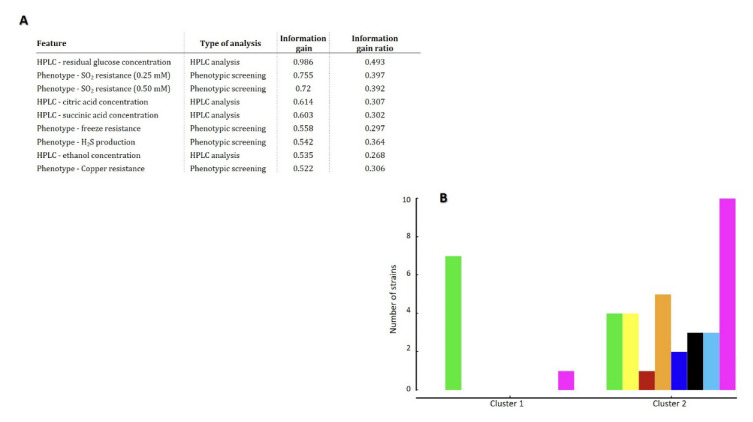
k-means cluster analysis of *T. delbrueckii* strains’ characterization. (**A**) Features (phenotypic tests and metabolites quantified by HPLC) mostly contributing for the division of strains into two clusters, in terms of information gain and information gain ration; (**B**) Number of strains categorized in the two clusters defined with k-means algorithm, colored according with their technological application or origin: ●—winemaking; ●—arboreal/soil; ●—food; ●—bread; ●—water; ●—clinical; ●—other beverages; ●—unknown origin.

To correlate phenotypic and metabolic data, understanding their differential contribution to the strain heterogeneity of *T. delbrueckii*, the established dataset was analyzed through the k-means clustering algorithm [89]. Using silhouette score [90], the algorithm identified 2 significantly distinct clusters (Figure 3B), composed of 8 and 32 strains, respectively. The contribution of all of the metabolic and phenotypic features to the establishment of the two distinct groups was evaluated and weighted (Figure 3A).

Results show an equal contribution of both experimental methodologies (HPLC metabolites quantification and phenotypic screening), revealing that the final concentrations of glucose, ethanol and citric and succinic acids, together with the resistance to SO_2_, copper and to the freezing process, were the features most relevant to explain this differentiation. Interestingly, cluster 1 was composed almost entirely by arboreal/soil strains, with the exception of the commercial wine strain T30, that once again showed different patterns from the remaining wine strains. Cluster 2 incorporated the remaining strains, that showed characteristics shared by the remaining wine strains.

Our next goal was to construct a computation model robust enough to predict the *T. delbrueckii* biotechnological potential, based on its phenotypic and metabolic profiles. Eleven predictive models were used, and their performance was evaluated in terms of AUC, classification accuracy, F1 score, precision and recall (Supplementary Materials, Table S2). Neural networks were revealed as the classifier with the best performance metrics, and were selected for further analysis. Table 2 shows the confusion matrix obtained using the neural networks’ classification of the dataset, after 10-fold cross validation. The AUC score of the obtained predictions was 0.718, which is considered as moderately high [41], and well above that of an arbitrary classification (AUC = 0.5; the AUC score of a perfect classifier is 1.0). The confusion matrix obtained from cross-validation classifications shows that the majority of the strains from the larger groups were correctly assigned to the respective technological group (highlighted in red in Table 2), even though some incorrect classifications were obtained between the groups of arboreal/soil and wine groups, which are in line with the shared features observed before in other analyses. Poor results were obtained for the remaining groups, which is due to the corresponding small number of isolates.

These results demonstrate the potential of these models to classify yeasts based on their phenotypic and metabolic data, together with the capacity to predict strains capable of being used in winemaking or in applications related to the environmental/ecological field, using only the proposed experimental tests described in Figure 3A. Similar results were obtained by us before, using the same type of models, to successfully predict the biotechnological potential of *S. cerevisiae* [30,91].

## 4. Conclusions

In conclusion, the present work represents the first detailed and extended phenotypic and metabolic characterization of a large collection of *T. delbrueckii* strains, comprising physiological tests and analytical determinations, mainly focusing on tests with biotechnological potential for winemaking and bread production. Our results demonstrate also the usefulness of computational approaches to describe relevant features among groups of *T. delbrueckii* strains that might occur as adaptive mechanisms in specific environments, mainly directed to winemaking or ecological applications, once the two large groups of strains analyzed in this study were obtained in these environments. Our computational models failed to correlate phenotypic features with the geographical locations from where the strains were obtained, detailing, however, the strong correlation between the phenotypic heterogeneity and the strains’ technological group, which is in accordance with what was observed for other yeast species [30]. With the knowledge arising from this work, together with the models proposed, predictions about the biotechnological potential of *T. delbrueckii* can be drawn based on the experimental data, and, in this way, can facilitate the selection of candidate strains to be used commercially.

## Figures and Tables

**Table 1:** Number of strains belonging to different phenotypic classes, as result of the screening comprising 31 tests, regarding values of optical density (Class 0: DO_640_ = 0.1; Class 1: 0.2 < DO_640_ < 0.4; Class 2: 0.5 < DO_640_ < 1.0; Class 3: DO_640_ > 1.0), killer phenotype (Class 0: neutral; Class 1: killer or sensitive phenotype), change of medium color (arbutin and BiGGY media), halo size (esculin medium) and cell viability after freezing.

Phenotypic Test	Type of Medium	Phenotypic Classes			
		0	1	2	3
15 °C	Liquid (synthetic must)	3	3	26	8
25 °C	Liquid (synthetic must)	0	0	0	40
30 °C	Liquid (synthetic must)	0	0	0	40
37 °C	Liquid (synthetic must)	35	1	2	2
Ethanol 5% (*v*/*v*)	Liquid (synthetic must)	1	1	2	36
Ethanol 10% (*v*/*v*)	Liquid (synthetic must)	21	11	5	3
Ethanol 14% (*v*/*v*)	Liquid (synthetic must)	37	3	0	2
Ethanol 18% (*v*/*v*)	Liquid (synthetic must)	38	2	0	0
Glucose (200 g/L)	Liquid (synthetic must)	0	0	0	40
Fructose (200 g/L)	Liquid (synthetic must)	0	0	2	38
Sucrose (200 g/L)	Liquid (synthetic must)	0	0	1	39
Maltose (200 g/L)	Liquid (synthetic must)	0	8	25	7
NaCl (1.5 M)	Liquid (synthetic must)	2	8	26	4
KCl (1 M)	Liquid (synthetic must)	0	0	1	39
H_2_O_2_ (2 mM)	Liquid (synthetic must)	13	0	1	26
CuSO_4_ (5 mM)	Liquid (synthetic must)	19	11	8	2
Fluconazole (0.5 mg/mL)	Liquid (synthetic must)	0	0	9	31
Myclobutanil (0.5 mg/mL)	Liquid (synthetic must)	0	4	26	10
Metalaxyl (0.5 mg/mL)	Liquid (synthetic must)	0	0	0	40
Tebuconazole (0.5 mg/mL)	Liquid (synthetic must)	0	2	19	19
*β*-glucosidase activity	Solid (Arbutin Agar)	0	19	18	3
	Solid (Bile Esculin Agar)	2	12	24	2
H_2_S production	Solid (BiGGY Agar)	4	2	10	24
Ethanol 12% (*v*/*v*)	Solid (MEA)	3	15	13	9
Ethanol 12% (*v*/*v*) + 0.25 mM SO_2_	Solid (MEA)	15	11	7	7
Ethanol 12% (*v*/*v*) + 0.5 mM SO_2_	Solid (MEA)	18	10	5	7
Ethanol 12% (*v*/*v*) + 1.0 mM SO_2_	Solid (MEA)	23	8	5	4
Ethanol 12% (*v*/*v*) + 1.5 mM SO_2_	Solid (MEA)	28	3	6	3
Killer activity—killer phenotype	Solid (YPD-MB Agar)	39	1	-	-
Killer activity—sensitive phenotype	Solid (YPD-MB Agar)	37	3	-	-
Freeze resistance	Liquid (LF)	7	6	10	17

**Table 2:** Confusion matrix indicating the technological group of 40 *T. delbrueckii* strains versus the technological group predicted using neural networks (AUC = 0.718). Correct predictions are highlighted in red.

		Predicted Technological Group								
		Arboreal/Soil	Bread	Clinical	Food	Other Beverages	Unknown	Water	Wine	Total
Real technological group	Arboreal/Soil	**6**	0	0	0	1	0	0	4	11
	Bread	0	**3**	0	0	0	0	0	1	4
	Clinical	0	0	0	0	0	0	0	1	1
	Food	0	0	0	**1**	1	0	1	2	5
	Other beverages	0	1	0	0	0	1	0	0	2
	Unknown	1	1	0	0	0	0	1	0	3
	Water	0	0	0	2	0	0	0	1	3
	Wine	4	0	0	0	0	0	1	**6**	11
	Total	11	5	0	3	2	1	3	15	40

## Data Availability

Not applicable.

## Supplementary Materials

The following supporting information can be downloaded at: https://www.mdpi.com/article/10.3390/jof8060569/s1, Table S1: Technological and geographical origins of the 40 *Torulaspora delbrueckii* strains used in this study; Table S2: Performance scores of the predictive classification algorithms after 10-fold cross validation, using the Orange data mining software for *T. delbrueckii* technological groups classification; Figure S1: Phenotypic data obtained after characterization of the 40 *Torulaspora delbrueckii* strains; Figure S2: Principal component analysis of phenotypic data of 40 *T. delbrueckii* strains, showing PC1 versus PC3; Figure S3: Heatmap of phenotypic data, after the characterization of the 40 *Torulaspora delbrueckii* strains; Figure S4: HPLC analysis of 40 *T. delbrueckii* strains after 192 h of fermentation; Figure S5: Principal component analysis of metabolic data of 40 *T. delbrueckii* strains, showing PC1 versus PC3.
