# Supplementary material for: Torulaspora delbrueckii Phenotypic and Metabolic Profiling towards Its Biotechnological Exploitation
Source: J Fungi (Basel). 2022 May 26;8(6):569. doi: 10.3390/jof8060569 (PMC9225199; doi:10.3390/jof8060569)
Supplement: Supplementary file 1 [file jof-08-00569-s001.zip › Supplementary Figure S1.pdf]

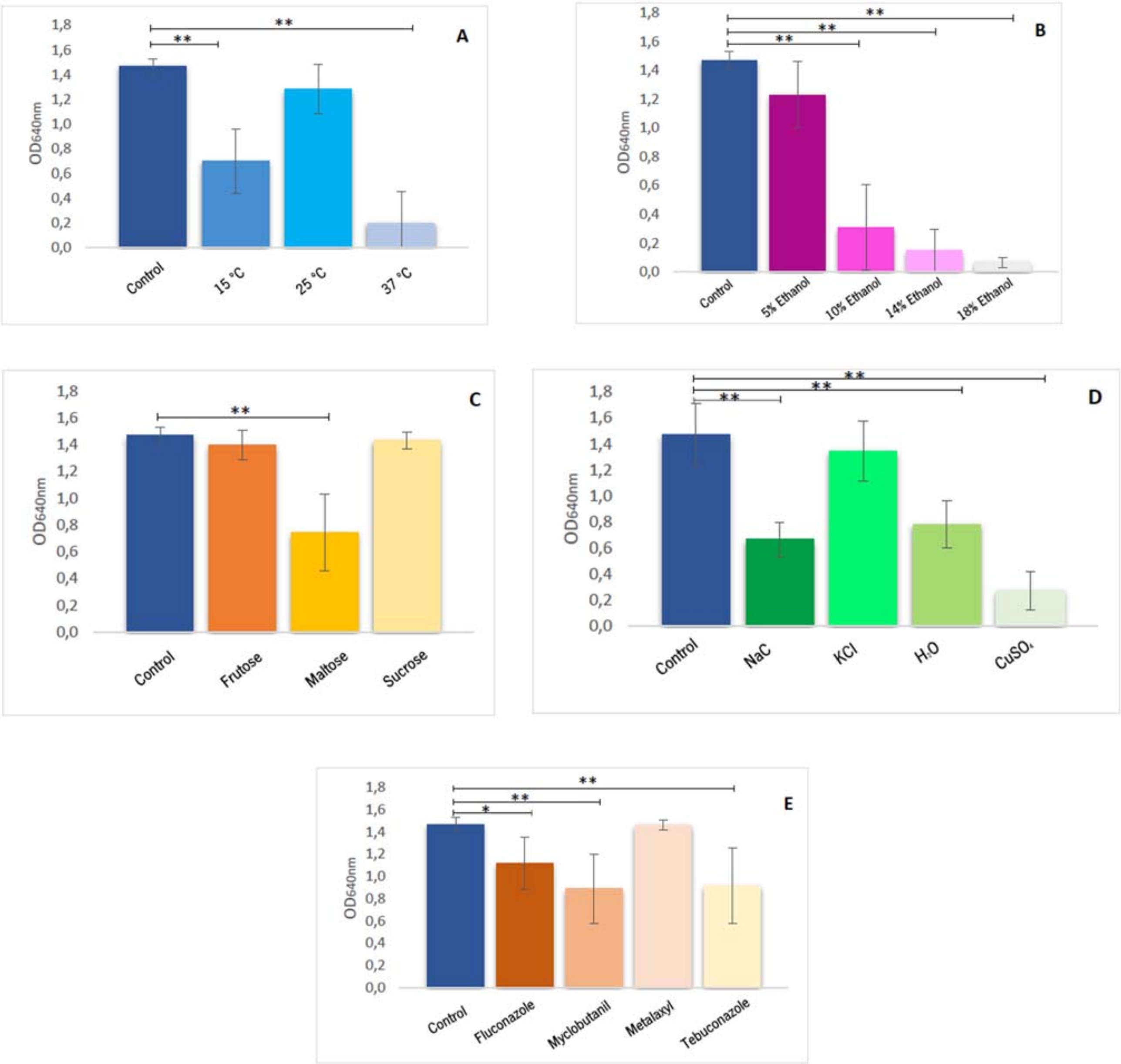

Supplementary Figure S1: Phenotypic data obtained after characterization of the 40 *Torulaspora delbrueckii* strains. Bars represent average of optical density determined for all isolates. **A** – comparison between temperatures; **B –** comparison between different concentrations of ethanol; **C** - effect of different carbon sources in the strains' growth; **D** – evaluation of stressful conditions; **E** – effect of fungicides in *T. delbrueckii* growth. Statistical analysis was performed applying one-way ANOVA (Fisher LSD Method), and comparing each test against the control (growth at 30 ˚C using glucose as carbon source). * $p<0.005$; **$p<0.001$
