# Supplementary material for: Torulaspora delbrueckii Phenotypic and Metabolic Profiling towards Its Biotechnological Exploitation
Source: J Fungi (Basel). 2022 May 26;8(6):569. doi: 10.3390/jof8060569 (PMC9225199; doi:10.3390/jof8060569)
Supplement: Supplementary file 1 [file jof-08-00569-s001.zip › Supplementary Figure S2.pdf]

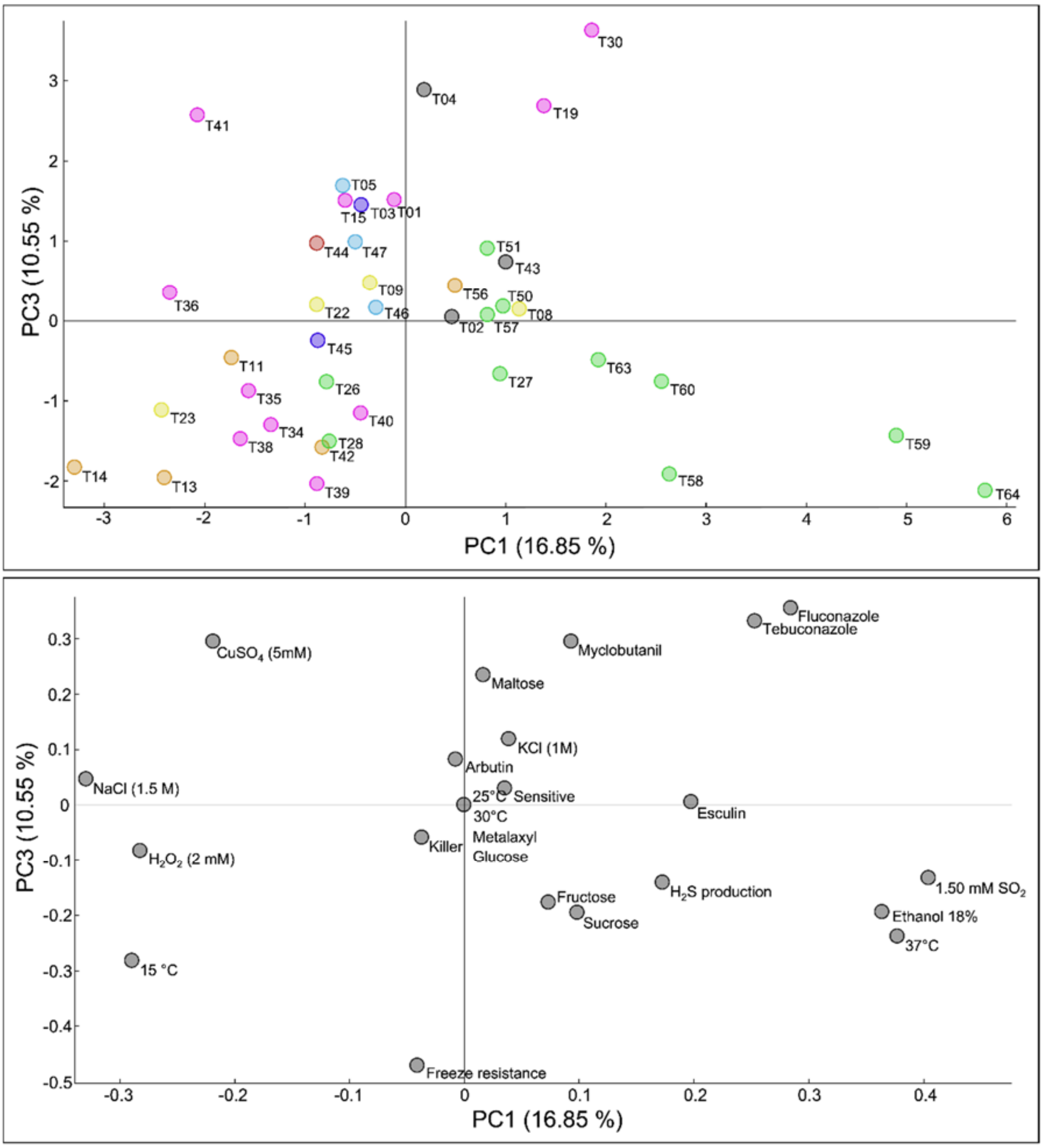

Supplementary Figure S2: Principal component analysis of phenotypic data of 40 *T. delbrueckii* strains, showing PC1 *versus* PC3.

A: Scores – 40 strains distribution. Colours represent the technological application or origin of the strains: ● – winemaking, ● - arboreal/soil, ● – food, ● – bread, ● – water, ● – clinical, ● – other beverages, ● – unknown origin.

B: Loadings – 24 phenotypic tests.
