# Supplementary material for: Torulaspora delbrueckii Phenotypic and Metabolic Profiling towards Its Biotechnological Exploitation
Source: J Fungi (Basel). 2022 May 26;8(6):569. doi: 10.3390/jof8060569 (PMC9225199; doi:10.3390/jof8060569)
Supplement: Supplementary file 1 [file jof-08-00569-s001.zip › Supplementary Figure S3.pdf]

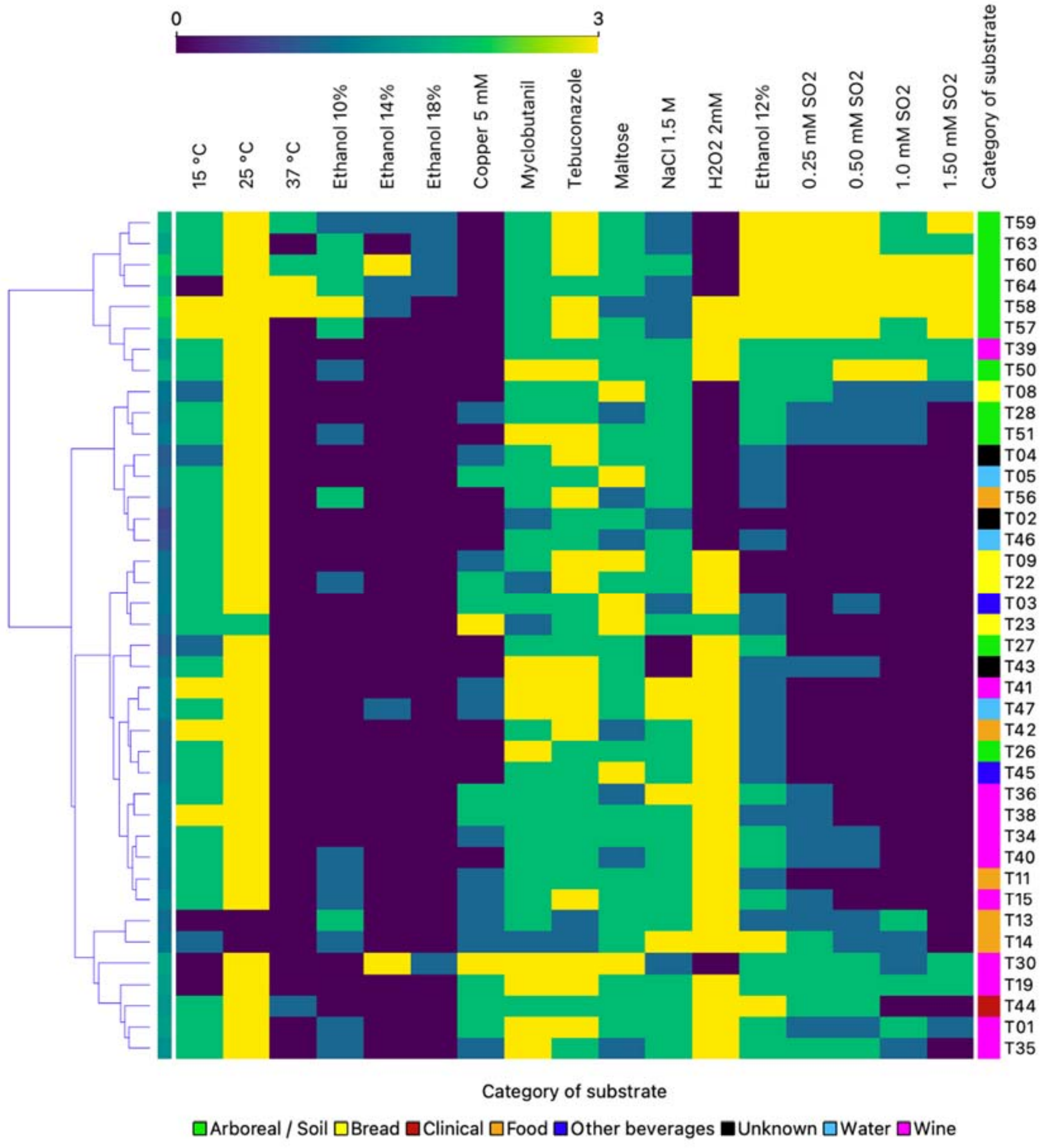

Supplementary Figure S3: Heatmap of phenotypic data, after the characterization of the 40 *Torulaspora delbrueckii strains* using 17 tests. Only the tests contributing with higher variability were considered in the analysis.
