# Supplementary material for: Torulaspora delbrueckii Phenotypic and Metabolic Profiling towards Its Biotechnological Exploitation
Source: J Fungi (Basel). 2022 May 26;8(6):569. doi: 10.3390/jof8060569 (PMC9225199; doi:10.3390/jof8060569)
Supplement: Supplementary file 1 [file jof-08-00569-s001.zip › Supplementary Figure S4.pdf]

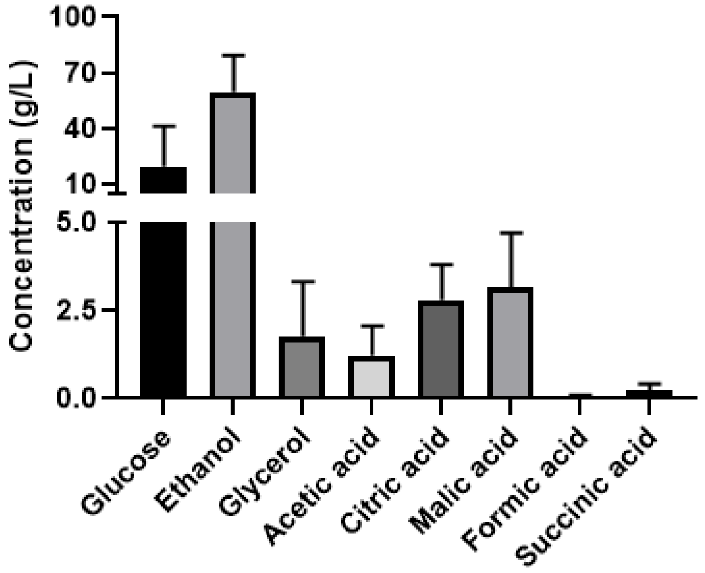

Supplementary Figure S4 – HPLC analysis of 40 *T. delbrueckii* strains after 192h of fermentation. Bars represent average concentrations (g/L) of three replicates, plus standard deviations.
