# Supplementary material for: Torulaspora delbrueckii Phenotypic and Metabolic Profiling towards Its Biotechnological Exploitation
Source: J Fungi (Basel). 2022 May 26;8(6):569. doi: 10.3390/jof8060569 (PMC9225199; doi:10.3390/jof8060569)
Supplement: Supplementary file 1 [file jof-08-00569-s001.zip › Supplementary Figure S5.pdf]

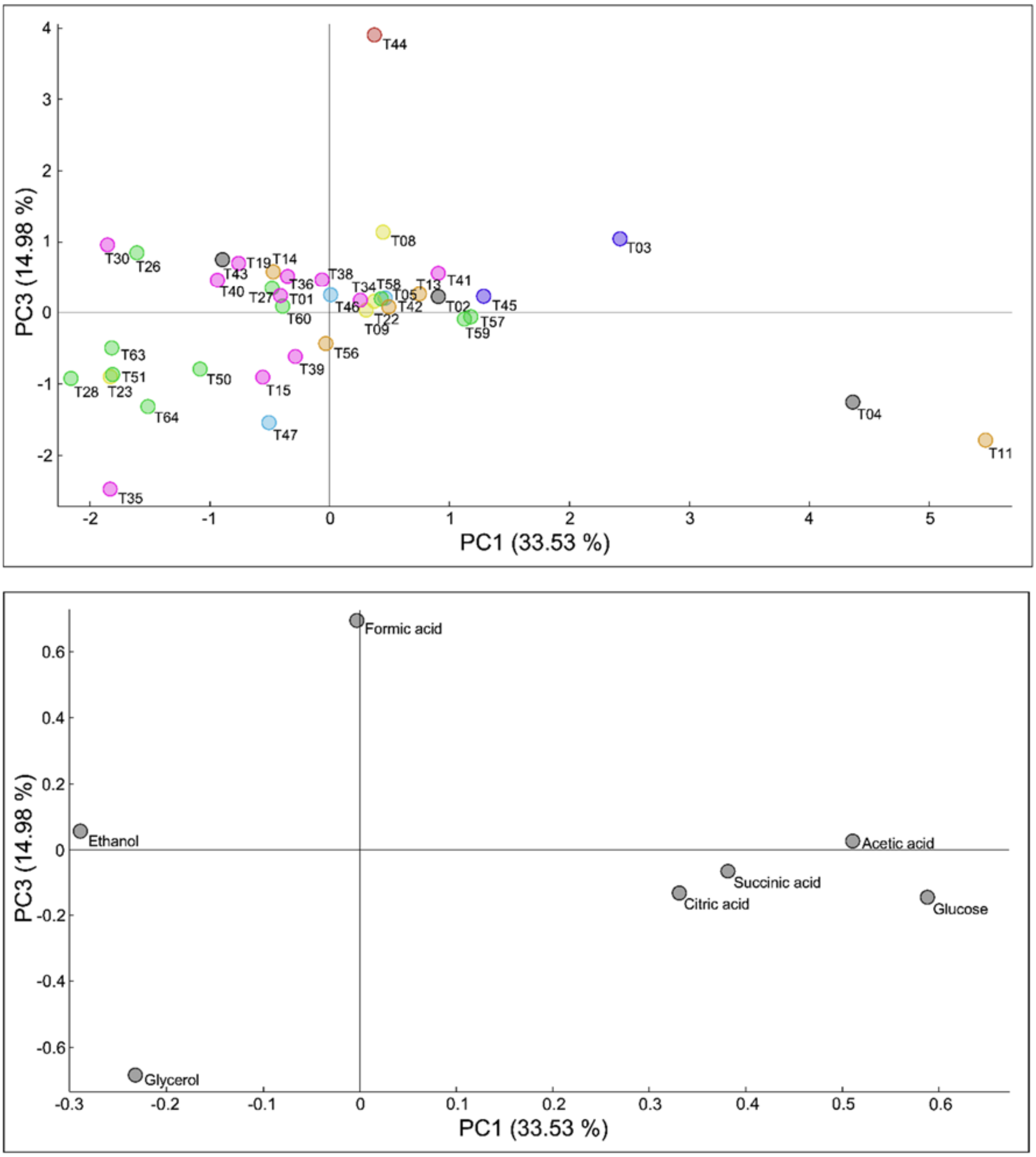

Supplementary Figure S5: Principal component analysis of metabolic data of 40 *T. delbrueckii* strains, showing PC1 *versus* PC3.
A: Scores – 40 strains distribution. Colours represent the technological application or origin of the strains: ● – winemaking, ● - arboreal/soil, ● – food, ● – bread, ● – water, ● – clinical, ● – other beverages, ● – unknown origin.
B: Loadings – concentration of 8 metabolites obtained by HPLC analysis, after 192h of fermentation
